# Do Omega-3/6 Fatty Acids Have a Therapeutic Role in Children and Young People with ADHD?

**DOI:** 10.1155/2017/6285218

**Published:** 2017-08-30

**Authors:** E. Derbyshire

**Affiliations:** Nutritional Insight Ltd, Surrey, UK

## Abstract

**Background:**

Attention deficit hyperactivity disorder (ADHD) is a debilitating behavioural disorder affecting daily ability to function, learn, and interact with peers. This publication assesses the role of omega-3/6 fatty acids in the treatment and management of ADHD.

**Methods:**

A systematic review of 16 randomised controlled trials was undertaken. Trials included a total of 1,514 children and young people with ADHD who were allocated to take an omega-3/6 intervention, or a placebo.

**Results:**

Of the studies identified, 13 reported favourable benefits on ADHD symptoms including improvements in hyperactivity, impulsivity, attention, visual learning, word reading, and working/short-term memory. Four studies used supplements containing a 9 : 3 : 1 ratio of eicosapentaenoic acid : docosahexaenoic acid : gamma linolenic acid which appeared effective at improving erythrocyte levels. Supplementation with this ratio of fatty acids also showed promise as an adjunctive therapy to traditional medications, lowering the dose and improving the compliance with medications such as methylphenidate.

**Conclusion:**

ADHD is a frequent and debilitating childhood condition. Given disparaging feelings towards psychostimulant medications, omega-3/6 fatty acids offer great promise as a suitable adjunctive therapy for ADHD.

## 1. Background

Attention deficit hyperactivity disorder (ADHD) is a common child-onset neurodevelopmental disorder occurring in children, adolescents, and adults, with an estimated prevalence of 5 to 7 per cent across cultures [1]. ADHD tends to be more common in boys than girls and is highly heritable, with pre- and perinatal factors also being implicated, although its definite cause remains unknown [2]. Although the rate of ADHD declines with age, at least half of children with the disorder will go on to have symptoms in adulthood [3]. The condition can impact heavily on mental health and education, lead to antisocial behaviour and personal dysfunction, and increase mortality risk [4]. Medications used to treat ADHD typically include methylphenidate (MPH; also, known as Ritalin), amphetamine, and atomoxetine which typically assume that there is a dopamine/norepinephrine deficit, although the aetiology of this condition is more complex [5]. Whilst MPH may ameliorate some comorbidities [6] it has been found to be ineffective in eliminating symptoms in 50 per cent of cases [7, 8]. Parents also appear to be concerned about the long-term effects of their children using medications such as MPH [9].

Long-chain polyunsaturated fatty acids (LCPUFA) and particularly omega-3 fatty acids have been under the spotlight for decades. They are key regulators of brain neurotransmission, neurogenesis, and neuroinflammation, all having an important role in the prevention and treatment of psychological and behavioural dysfunction disorders [10]. Eicosapentaenoic acid (EPA) and docosahexaenoic acid (DHA) are two fatty acids that are highly concentrated in the brain, exhibiting antioxidative, anti-inflammatory, and antiapoptotic effects, with these contributing to neuron protection [11].

The omega-6 fatty acid gamma linolenic acid (GLA) is also important in the generation of arachidonic acid (ARA) which is abundantly present in in the brain [12, 13]. A recent meta-analysis found that combinations of omega-3 and omega-6 fatty acids (EPA and GLA) helped to improve symptoms of inattention in children with ADHD [14]. Brain lipids within cell membranes also act as signalling mediums, supporting neurotransmitter function with omega-3 fatty acids thought to play a key role in this which may help in the prevention of anxiety disorders [15]. Laboratory research has also identified that omega-3 fatty acids may act in a similar way to “antipsychotics,” possibly by acting on brain receptors and helping to restore oxidative balance [16].

Omega-3 deficiencies have been found to alter dopaminergic and serotonergic systems, potentially modifying cerebral receptors in specific regions of the brain [17]. EPA and DHA are regarded as “essential fatty acids (EFAs)” that need to be obtained from food or supplement sources as they cannot be made in sufficient amounts by the human body [11]. The ratio of fatty acids (omega-6 : omega-3) which complete for the same enzyme pathways can also influence neurotransmission and prostaglandin formation, both of which are crucial in the maintenance of normal brain function [18, 19]. Furthermore, as the storage of the omega-3 fatty acids is limited, a continual exogenous supply is needed to obtain suitable levels [20].

A number of studies have measured LCPUFA status in individuals with ADHD. One study conducted on young adults (22.3 to 24.3 years) found the proportion of omega-3 fatty acids was significantly lower in the plasma phospholipids and red blood cells of ADHD participants compared with controls, whilst levels of saturated fatty acids were higher [21]. Another investigation found that whilst teenagers with ADHD consume similar amounts of omega-3 and omega-6 fatty acids to controls, their DHA status was significantly lower, indicating metabolic differences in fatty acid handling in those with ADHD [22]. Similarly, another trial showed that the proportions of saturated and polyunsaturated fatty acids were higher and lower, respectively, in paediatric patients with ADHD, compared with controls again indicating differences in lipid profiles [23]. Further meta-analytical evidence has concluded that children and young people with ADHD have elevated ratios of blood omega-6/3 indicating disturbances in fatty acid metabolism in these individuals [24].

Given that the human brain is nearly 60 per cent fat and the central role that EFAs have to play in the structure, synthesis, and functions of brain neurotransmitters [25], the present article evaluates evidence on whether LCPUFAs have a therapeutic role in the management of ADHD. Particular focus will be given on their potential effects in the management of ADHD along with their role as an adjunctive therapy.

## 2. Methods

### 2.1. Approach

The National Centre for Biotechnology Information (NCBI) search engine (PubMed) was used to extract relevant publications. English-language, human, randomised controlled trials (RCTs) published between 2001 and March 2017 were included. Data files were extracted from the NCBI collection depository and imported into Covidence software used to create systematic reviews.

### 2.2. Exclusion/Inclusion Criteria

Publications were excluded if they were not a RCT, did not use participants with ADHD, or were conducted on older adults with ADHD. For inclusion studies needed to be conducted on children or young people (up to 18 years of age), participants were considered to have ADHD at baseline and were taking an omega-3/6 supplement, including EPA, DHA, or GLA. Publications were further included if the full text was available or could be purchased.

The search terms “attention deficit hyperactivity disorder” or “ADHD” were combined with “long-chain n-3 fatty acids”, “omega-3/6 fatty acids”, “docosahexaenoic acid”, “eicosapentaenoic acid”, and “gamma linoleic acid”. Data extracted from each article included (1) author(s) and country of research, (2) subjects (gender, number of participants), (3) mean age, (4) study design and methods, (5) dose of supplement, and (6) main findings.

## 3. Results

**Figure 1:**
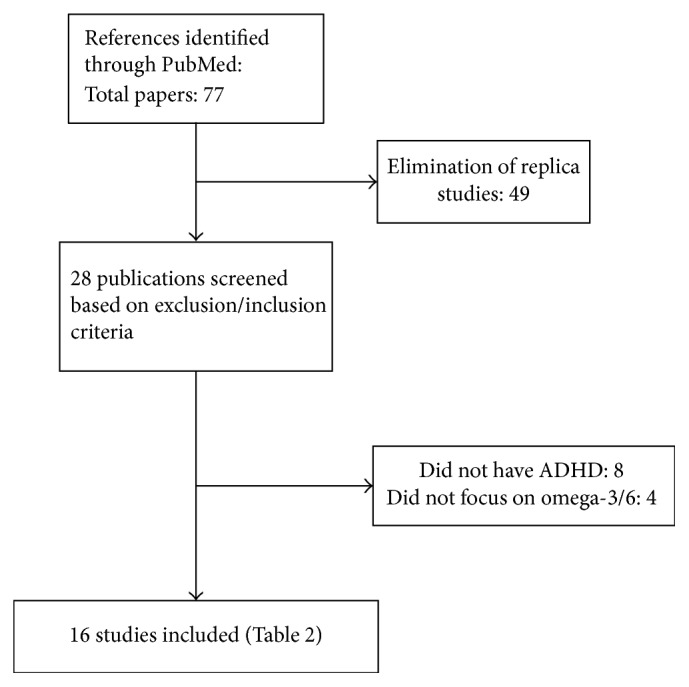
Algorithm of qualifying publications.

The NCBI search identified 77 papers. After a further adjustment for replica papers, 28 articles remained for assessment. Of these, 12 papers were discarded after reviewing the abstracts and article content as they did not meet the inclusion criteria. This left 16 RCTs for general review.  Figure 1 shows the algorithm of qualifying publications. Of these, one study was conducted in the United Kingdom, five in Europe, one in the United States, one in Mexico, two in Australasia, four in Asia, and two in the Middle East.

### 3.1. Definitions

As shown in Table 1, all of the publications identified included children or young people with ADHD at baseline. Most studies diagnosed ADHD according to the Diagnostic and Statistical Manual of Mental Disorders, 4th Edition (DSM-IV) criteria. Others used methods such as the Conners' Parent Rating Scale (CPRS) and parent-reported learning difficulties [26–28]. Some studies focused more specifically on certain ADHD subtype. For example, Widenhorn-Müller et al. (2014) included the inattentive and hyperactive/impulsive subtypes within the trial whilst Voigt et al. (2001) studied those with oppositional defiant or conduct disorders. Other trials used adapted parental/researcher screening tools alongside the DSM-IV [29, 30].

### 3.2. Omega Fatty Acids

A total of 16 RCTs studied interrelationships between combinations of omega-3/6 fatty acids and ADHD symptoms (Table 2). Of these 13 reported beneficial effects, though the levels of effect appeared to depend on the dose of the intervention, ratio of the fatty acids, quality of the RCT, and ADHD subtype under investigation.

One of the most recent studies found that children (aged 6 to 12 years) receiving omega-3/6 fatty acids (Equazen) providing 558 mg EPA, 174 mg DHA, and 60 mg GLA in a 9 : 3 : 1 ratio over a period of 12 months did not need such a high dose of MPH to manage and reduce their ADHD symptoms (0.8 mg/kg/day versus 1.0 mg/kg/day). The completion rate was also higher in this group, whilst the withdrawal rate and the incidence of adverse events were significantly lower. These findings indicate that omega-3/6 fatty acids may act as a useful adjunctive therapy to MPH, helping to improve tolerability, dosing, and adherence [31].

A 12-week RCT comprised of 76 male adolescents with ADHD using a similar dose of fatty acids found that supplementation improved blood levels of EPA, DHA, and total omega-3 fatty acids, though no effects on aggression, impulsivity, or anxiety were seen, possibly due to the smaller study sample size and shorter study length of this trial [32]. Two other trials have been undertaken using a similar 9 : 3 : 1 ratio of EPA, DHA, and GLA, respectively [33, 34]. This work was the first to trial omega-3/6 fatty acids finding that 1 in 8 patients benefited and experienced a reduction of more than 50% of ADHD symptoms, with strongest results seen amongst boys and those with ADHD inattentive subtype [34]. Later research by the same team of scientists found that omega-3/6 supplementation significantly improved the fatty acid composition amongst study “responders,” that is, those with more than a 25 per cent reduction in ADHD symptoms [33].

Three studies used functional foods providing LC3PUFA. In a double-blind RCT, the ingestion of 10 g margarine daily providing 650 mg EPA/DHA improved parent-rated attention in children with ADHD after 16 weeks in 8–14-year-olds who continued with their usual medication. Another trial using “omega eggs” providing EPA and DHA found that daily consumption by 7–12-year-olds over 3 months significantly improved visual acuity and the red blood cell fatty acid profile of children with lower intelligent quotients or ADHD, indicating that the DHA content of ordinary eggs may not be sufficient [27]. Another work giving 6- to 12-year-olds ADHD DHA-enriched foods showed that ADHD symptoms did not improve though there were some significant improvements in short-term memory and errors of continuous performance [35].

Three studies concluded that there were limited associations between omega-3/6 fatty acid supplementation and ADHD outcomes. In one study, Conners' Parent and Teacher Rating Scale was not regarded as being sensitive enough to detect small improvements in the behaviour of male adolescents [32]. Another work found that a supplement providing 480 mg of linoleic acid and 120 mg of *α*-linolenic acid ameliorated some ADHD symptoms amongst 7–13-year-olds, although no significant differences were found, possibly because children were unmedicated [30]. Earlier work providing 6- to 12-year-olds with 345 mg DHA over 4 months did not find this to ameliorate ADHD, indicating that a longer trial period and inclusion of arachidonic acid may have been needed [36].

Remaining studies showed general benefits. An Australian study found that children with ADHD who had increased erythrocyte EPA + DHA levels had significantly improved spelling and attention and reduced oppositional behaviour, hyperactivity, and cognitive problems [28]. An omega-3 fatty acid mix taken over 16 weeks by German children aged 6 to 12 years also increased EPA + DHA erythrocyte levels and improved working memory but had no other effects on behaviour [37]. A short 8-week trial reported significant improvements in hyperactivity scores after supplementation with EPA + DHA [38]. In a trial where children had been taking MPH, supplementation with omega-3 and omega-6 fatty acids in the ratio of 1.6 : 1 led to significant improvements in inattention and impulsiveness, along with cooperation with parents and teachers after 6 months, indicating this was a safe and effective adjunctive therapy [29].

Other trials showed findings to be more prominent in certain subgroups. For example, a large trial of 200 children found that supplementation with phosphatidylserine-omega-3 reduced ADHD symptoms in a subgroup of hyperactive-impulsive, emotionally and behaviourally dysregulated ADHD children compared with the placebo [39]. Another work found that erythrocyte DHA levels increased after 4 months of supplementation with 4 capsules daily providing either (1) 108 mg DHA and 1109 mg EPA, (2) 1032 mg DHA and 264 mg EPA, or (3) 1467 mg of linoleic acid [28]. The study also found that higher doses of DHA helped to improve the literacy and behaviour in children with ADHD, particularly in a subgroup with learning difficulties [28]. Norwegian work showed that 0.5 g EPA after 15 weeks improved symptoms in two ADHD subgroups: positional and less hyperactive/impulsive children [40].

## 4. Discussion

The aetiology of ADHD is complex and multifactorial though diet, nutrition, and abnormalities in the metabolism of LCPUFA are thought to have underlying roles [21, 22]. The present review has shown that omega-3 and omega-6 fatty acids have an important role to play in the management of ADHD. Previous work has shown that the tolerability of omega-3 fatty acids given to individuals with ADHD is high with only mild side effects reported such as incidental nose bleeds and gastrointestinal discomfort [42]. Severe side effects have not been documented and these minor complaints are regarded as being less severe than methylphenidate side effects [42].

Taken together, a growing body of clinically proven evidence suggests that dietary supplementation using omega-3/6 PUFAs may help to augment conventional ADHD treatments. Research carried out in Mexico at the National Health Institute with children prescribed with MPH and taking omega-3/6 fatty acids found that they required lower doses of the prescription medicine and experienced fewer medication-related side effects [31]. Similarly, other work has shown that omega-3/6 supplementation reduced behavioural and learning difficulties in children with ADHD that was refractory to MPH treatment alone [29]. Another RCT concluded that EPA was a safe complementary treatment option in omega-3 deficient ADHD children, with scope to benefit ADHD subgroups who are less responsive to stimulant treatments [40]. A recent review of 25 clinical trials has also concluded that two patients groups, in particular, could benefit from omega-3 fatty acids. The first is those with mild ADHD where omega-3 supplements could replace stimulant medications. The second is those with severe ADHD where omega-3 supplements could reduce the amount of stimulant medication being used, in turn, potentially reducing symptoms from the medications side effects [42].

These studies are further supported by evidence from meta-analytical studies. Evidence collated from ten trials comprised of 700 children has shown that omega-3 supplementation, with higher doses of EPA had modest effects in the treatment of ADHD, indicating potential roles in augmenting traditional pharmacological treatments, whilst providing an option for families who may decline other psychopharmacologic options [43]. An earlier meta-analysis also concluded that omega-3 fatty acids offer promise as a possible supplement to traditional therapies [44]. Interestingly, a series of interviews about treatment experiences showed that over half (52%) of parents expressed initial reluctance towards psychostimulants. Once psychostimulants were used by children and adolescents with ADHD, 73% concurrently used other treatments [45]. These findings indicate that parents are concerned about their children using psychostimulants and are looking for accompanying treatment options.

In terms of study outcomes, most focused on ADHD symptoms. Whilst reduced hyperactivity and impulsivity were reported in most studies [26, 29, 31, 38, 40], other outcomes such as improved attention [41], visual acuity [27], improved word reading [28], and working/short-term memory were also observed [35, 37]. These findings indicate that LCPUFA supplementation has far-reaching effects, having additional benefits for learning. A recent 6-month 2-phase randomised trial with 154 children aged 9 and 10 years showed the omega-3/6 fatty acid supplementation improved reading ability in mainstream children and improved cognitive measures in children with attention problems, defined as those with ADHD symptom scores above the median [46].

With regards to dose a 9 : 3 : 1 ratio of EPA (558 mg) to DHA (174 mg) and GLA (60 mg) was used in four studies [31–34]. In the largest and longest studies, this was associated with improved hyperactivity and impulsivity subscores [31] and reduced ADHD symptoms [33], especially in the inattentive ADHD subtype and those with comorbid neurodevelopment disorders [34]. Other work using 635 mg EPA and 195 mg DHA also led to significant improvements in ADHD scores [38]. Studies using lower doses (345 mg DHA) tended not to yield significant findings in terms of ADHD symptoms [36]. Taken together, it appears that higher doses of fatty acids are needed to generate measurable effects. The ratio of omega-6 to omega-3 in studies and especially the ARA/DHA ratio may also have impacted on study outcomes, as this is regarded as being important for membrane fluidity [47].

Inconsistencies of lack of findings in some trials may have been attributed to interventions being too short. As erythrocytes survive in the body for 120 days, supplementation trials shorter than 12 weeks (84 days) may not be sufficient enough to detect changes in LCPUFA compositions [32]. Equally, as the turnover of fatty acids in the brain is thought to be rather low in 6- to 12-year-olds, longer periods of supplementation and/or higher doses may be needed to modify the fatty acid content of the central nervous system [36]. It should also be considered that some studies used different tools to assess ADHD symptoms, measures of attention, and scales of hyperactivity, some of which may be more sensitive than others. On a final note, it should be considered that the Diagnostic and Statistical Manual of Mental Disorders, 5th Edition (DSM-5) is now out which includes new diagnostic groups such as “disruptive mood regulation” which have potential to be applied in future studies [48]. This could possibly increase prevalence rates of mental health disorders in future trials [48]. Continued research using the latest DSM-5 criteria, along with larger and longer interventions (more than 12 weeks), is now needed.

## 5. Conclusion

In conclusion, ADHD is a debilitating neurodevelopmental disorder that can impact heavily on children and young people's behaviour, mental health, education, and social/family lives. Whilst conventional medications have a role to play in the management of ADHD symptoms, new clinically trialled evidence indicates that omega-3/6 supplementation programmes can provide a promising adjunctive therapy, lowering the dose of psychopharmacologic medications needed and subsequently improving compliance with these. It also appears that parents are looking for complementary treatments for their children to use alongside traditional treatments.

## Figures and Tables

**Table 1:** Methods used to screen for ADHD.

Author	Definition used
Barragán et al. (2017)	ADHD of any subtype. Diagnosed according to the DSM-IV criteria and CGI-S scale.

Bos et al. (2015)	ADHD diagnosis confirmed by a trained researcher using the DISC-P.

Matsudaira et al. (2015)	ADHD diagnosis confirmed through a semi-structured interview based on the DSM-IV criteria.

Milte et al. (2015)	Diagnosis of ADHD or parent-rated symptoms >90th percentile on the CPRS and parent-reported learning difficulties.

Wu et al. (2015)	ADHD diagnosed according to DSM-IV and the Chinese version of CPRS. These rating scales about learning, attention, and behaviour were completed by the teachers and either parent(s) or guardians.

Widenhorn-Müller et al. (2014)	Met DSM-IV criteria for the ADHD combined subtype (hyperactive–inattentive) and the primarily inattentive or the hyperactive/impulsive subtype were included in the trial.

Manor et al. (2013)	Children were included if they had a score of at least 1.5 standard deviations above the normal for the patient's age and gender in the Teacher-Rated ADHD Rating Scale-IV School Version.

Hariri et al. (2012)	Conners' Abbreviated Questionnaires scores for hyperactivity were greater than 14.

Johnson et al. (2012)	Participants met DSM-IV criteria for a diagnosis of ADHD.

Milte et al. (2012)	Diagnosis of ADHD or parent-rated symptoms >90th percentile on the CPRS and parent-reported learning difficulties.

Perera et al. (2012)	All children in the program were clinically diagnosed using DSM-IV supported by positive scores in Swanson, Nolan, and Pelham version IV (SNAP) parent and teacher evaluation.

Gustafsson et al. (2010)	Clinical diagnosis of ADHD of combined type (fulfilling DSM-IV criteria A–E) with any neuropsychiatric comorbidity and who had been evaluated for pharmacological treatment.

Johnson et al. (2009)	Participants met DSM-IV criteria for a diagnosis of ADHD of any subtype, scoring at least 1.5 SD above the age norm for their diagnostic subtype using norms for the ADHD Rating Scale–IV–Parent Version.

Raz et al. (2009)	Parents were asked to present a formal ADHD diagnosis. The child performed a continuous performance test, while one of the parents filled in the essential fatty acids deficiency questionnaire and the DSM-IV questionnaire.

Hirayama et al. (2004)	Diagnosed or suspected as AD/HD according to DSM-IV and diagnostic interviews including behaviour observation by psychiatrists. In a strict sense, eight subjects might not be AD/HD according to the DSM-IV criteria, but two psychiatrists attending the summer camp strongly suspected them as AD/HD.

Voigt et al. (2001)	Previously been given a diagnosis of ADHD by a physician. Confirmatory diagnostic interview with a neurodevelopmental paediatrician to confirm responses to the telephone interview and to ensure that each met DSM-IV.

*Key.* ADHD, attention deficit hyperactivity disorder; CGI-S scale; Clinical Global Impressions-Severity scale; CPRS, Conners' Parent Rating Scale; DISC-P, Diagnostic Interview Schedule for Children-Parent Version; DSM-IV; Diagnostic and Statistical Manual of Mental Disorders.

**Table 2:** Information extracted from trials looking at LC3PUFAs and ADHD.

Reference and country	Subjects M/F and sample size	Mean age	Study design and methods	Dose of supplement	Main findings
[31] Mexico	90 children (60 M, 30 F)	6–12 years Mean age 8.27 years	12-month trial (unblinded); MPH, omega-3/6 or a combination	Equazen: 558 mg EPA, 174 mg DHA, and 60 mg GLA (9 : 3 : 1 ratio)	Significantly better scores on ADHD. Adverse events were numerically less frequent with omega-3/6 or MPH + omega-3/6 than MPH alone.

[41] Netherlands	40 boys with ADHD and 39 matched, typically developing controls	Aged 8–14 years	16-week trial	10 g of margarine daily, enriched with either 650 mg of EPA/DHA or placebo	EPA/DHA supplementation improved parent-rated attention in both children with ADHD and typically developing children. Phospholipid DHA level at follow-up was higher for children receiving EPA/DHA supplements than placebo.

[32] United Kingdom	76 M adolescents with ADHD	12–16 years, mean = 13.7 years	12-week trial	Equazen: 558 mg EPA, 174 mg DHA, and 60 mg GLA (9 : 3 : 1 ratio)	In the treatment group, supplementation enhanced EPA, DHA, and total omega-3 fatty acid levels.

[26] Australia	90 Australian children with ADHD symptoms higher than the 90th percentile on the Conners' Rating Scales	7 to 12 years	4-month crossover study evaluating literacy and behaviour up to 12 months	Supplements rich in EPA, DHA, or LA	Increased erythrocyte EPA + DHA was associated with improved spelling (*p* < 0.001) and attention (*p* < 0.001), reduced oppositional behaviour (*p* < 0.003), hyperactivity (*p* < 0.001), cognitive problems (*p* < 0.001), DSM-IV hyperactivity (*p* = 0.002), and DSM-IV inattention (*p* < 0.001).

[27] China	179 children with lower IQs or ADHD to receive	7 to 12 years	3-month trial: evaluated effects on visual acuity	Ordinary eggs or eggs rich in EPA and DHA	Both groups of children showed a significant improvement in visual acuity (*p* < 0.05); however, visual acuity in the study group was significantly better than that of the control group (*p* = 0.013).

[37] Germany	95 children diagnosed with ADHD according to DSM-IV criteria	6–12 years	16-week trial	Omega-3 fatty acid mix	Improved working memory correlated significantly with increased EPA, DHA, and decreased ARA.

[39] Israel	200 children diagnosed with ADHD	6–13 years	15-week trial followed by an open-label extension	300 mg PS-omega-3/day	Study results demonstrate that consumption of PS-omega-3 by children with ADHD, is safe and well tolerated, without any negative effect on body weight or growth.

[38] Malaysia	103 children	6–12 years	8-week trial	635 mg EPA, 195 mg DHA	Significant reduction in levels of CRP in the omega-3 group and significant increase in SOD and glutathione reductase. Significant improvement in ASQ-P score (measure of hyperactivity).

[33] Sweden	75 children and adolescents with DSM-IV ADHD	8–18 years	3-month trial. Omega-3/6 (Equazen) or placebo, followed by 3 months of open phase	Omega-3/6 (Equazen) or placebo Equazen: 558 mg EPA, 174 mg DHA, and 60 mg gamma linoleic acid (9 : 3 : 1 ratio)	Subjects with more than 25% reduction in ADHD symptoms were classified as responders. Compared to nonresponders, the 6-month responders had significantly greater n-3 increase at 3 months and decrease in n-6/n-3 ratio at 3 and 6 months (*p* < 0.05).

[28] Australia	90 Australian children with ADHD symptoms higher than the 90th percentile on the Conners' Rating Scales	7 to 12 years	4-month trial	Supplements rich in EPA, DHA, or safflower oil	Increased erythrocyte DHA was associated with improved word reading and lower parent ratings of oppositional behaviour. These effects were more evident in a subgroup of 17 children with learning difficulties.

[29] Sri Lanka	Children with ADHD *n* = 48 active group, *n* = 46 placebo	6–12 years	6-month trial	Capsule containing n3 and n6 (fish oil) and cold-pressed evening primrose oil	Statistically significant improvement was not found at 3 months of treatment between groups but was evident at 6 months of treatment (*p* < 0.05) with inattention, impulsiveness, and cooperation with parents and teachers.

[40] Norway	92 children with ADHD	7–12 years	15-week RCT	0.5 g EPA versus placebo	EPA improved CTRS, inattention/cognitive subscale (*p* = 0.04), but not Conners' total score.

[34] Sweden	75 children and adolescents with DSM-IV ADHD	8–18 years	3-month trial. Omega-3/6 (Equazen) or placebo, followed by 3 months of open phase	Equazen: 558 mg EPA, 174 mg DHA, and 60 mg GLA (9 : 3 : 1 ratio)	A subgroup of 26% responded with more than 25% reduction of ADHD symptoms and a drop of Clinical Global Impression scores to the near-normal range. After 6 months, 47% of all showed such improvement. Responders tended to have ADHD inattentive subtype and comorbid neurodevelopmental disorders.

[30] Israel	73 unmedicated children with a diagnosis of ADHD	7–13 years	7-week trial	480 mg LA, 120 mg ALA, placebo: 1000 mg of vitamin C	Both treatments ameliorated some of the symptoms, but no significant differences were found between the groups in any of the treatment effects.

[35] Japan	40 AD/HD (including eight AD/HD-suspected) children who were mostly without medication	6–12 years	2-month trial	Foods containing fish oil (fermented soybean milk, bread rolls, and steamed bread; 3.6 g DHA/week from these foods)	DHA-containing foods did not improve ADHD-related symptoms. Visual short-term memory and errors of commission (continuous performance) significantly improved in the control group compared with the changes over time in the DHA group.

[36] USA	63 children with ADHD, all receiving effective maintenance therapy with stimulant medication	6–12 years	4-month trial	345 mg DHA	No statistically significant improvement in any objective or subjective measure of ADHD symptoms.

*Key*. ADHD, attention deficit hyperactivity disorder; ALA, alpha-linolenic acid; ARA, arachidonic acid; CRP, C-reactive protein; CTRS, Connor Teacher Rating Scale; DHA, docosahexaenoic acid; DSM-IV; Diagnostic and Statistical Manual of Mental Disorders; EPA, eicosapentaenoic acid; F, female; GLA, gamma linoleic acid; LA, linoleic acid; M, male; MPH, methylphenidate; PS, phosphatidylserine; SOD, superoxide dismutase.

## Abbreviations

ARA: Arachidonic acid
ADHD: Attention deficit hyperactivity disorder
CPRS: Conners' Parent Rating Scale
CTRS: Connors' Teacher Rating Scale
DHA: Docosahexaenoic acid
DSM: Diagnostic and Statistical Manual of Mental Disorders
EFAs: Essential fatty acids
EPA: Eicosapentaenoic acid
GLA: Gamma linoleic acid
LCPUFA: Long-chain polyunsaturated fatty acids
MPH: Methylphenidate
RCT: Randomized controlled trial.
